# Estimated Health Outcomes and Costs Associated With Use of Monoclonal Antibodies for Prevention or Mitigation of SARS-CoV-2 Infections

**DOI:** 10.1001/jamanetworkopen.2022.5750

**Published:** 2022-04-05

**Authors:** Minah Park, Kelvin Bryan Tan, Shawn Vasoo, Borame L. Dickens, David Lye, Alex R. Cook

**Affiliations:** 1 Saw Swee Hock School of Public Health, National University of Singapore, Singapore; 2 Ministry of Health, Singapore; 3 National Centre for Infectious Diseases, Singapore

## Abstract

This economic evaluation investigates the health outcomes and costs associated with use of monoclonal antibodies for treatment of SARS-CoV-2 in 14 scenarios stratified by age, vaccination status, and source of infection.

## Introduction

Mass vaccination against COVID-19 has limited hospitalizations and deaths associated with the disease, but even in countries with excess supply of vaccines, substantial numbers of people remain unvaccinated and at risk of severe disease. The experience of high-income countries, such as Israel and Singapore, shows that serious illnesses requiring oxygen supplementation and intensive care will persist even as vaccination rates reach 80% of the population.^1^

Anti–SARS-CoV-2 monoclonal antibodies, including REGEN-COV (casirivimab and imdevimab), have been shown to prevent infection in household contacts^2^ and decrease risk of hospitalization or death related to COVID-19.^3^ Beyond the benefits to the individual, use of these treatments may also preserve scarce medical resources during outbreaks. Questions remain concerning whether monoclonal antibodies would best be used as prophylaxis, treatment, or a combination of prophylaxis and treatment. Therefore, we aimed to (1) assess potential health and cost benefits associated with using REGEN-COV as postexposure prophylaxis (PEP) in household contacts and for treating COVID-19 and (2) help policy makers with decisions about prioritization of REGEN-COV while supply is limited, using Singapore as a case study.

## Methods

The report of this economic evaluation follows the CHEERS reporting guideline. Our research involved the analysis of routinely collected, aggregated data for public health policy making, and ethical approval was not required, as advised by the Departmental Ethics Review Committee of the Saw Swee Hock School of Public Health at the National University of Singapore.

We identified 14 scenarios (eFigure in the Supplement) in which REGEN-COV was allocated to different groups of individuals at increased risk stratified by age, vaccination status, and source of infection (ie, household vs nonhousehold). Epidemiological and clinical characteristics of patients with COVID-19 were collated by the Ministry of Health and National Centre for Infectious Diseases of Singapore (eAppendix and eTables 1-3 in the Supplement).

Health outcomes included the number of patients with severe illnesses requiring oxygen supplementation, patients with critical illness admitted to the intensive care units, deaths due to COVID-19, and disability-adjusted life-years (DALYs). Economic outcomes were the overall cost of PEP and treatment with REGEN-COV, cost of hospitalization, and cost per DALY averted. We calculated net costs by subtracting baseline cost without REGEN-COV (ie, the status quo) from total cost with REGEN-COV. We performed sensitivity analyses by setting the relative risk reduction (RRR) of REGEN-COV to 31.6% and 87.1% (ie, the 95% CI bounds) instead of the point estimate of 70.4%.^3^ All analyses were conducted between September 2 and September 29, 2021.

## Results

All scenarios considered were cost-effective using the threshold of 1.15 gross national income^4^ per DALY; some were cost-saving. Treating recently diagnosed individuals and those aged 60 years and older with REGEN-COV was the most cost-saving, with a net cost saving of approximately US $340 000 for every 10 000 infections (Table). Using REGEN-COV as PEP in individuals exposed to infected family members was less cost-effective compared with using it to treat only infected individuals (cost saving of US $19 500 vs US $1200). Because all scenarios in which there were sufficient supplies were cost-effective, we considered which scenarios were most robust to the risk of exhausting supplies amid an epidemic wave. Preserving REGEN-COV for treatment of individuals aged 60 years and older was associated with the greatest decrease in DALYs and severe illnesses across a range of supply scenarios, although in scenarios with few available doses, use should be restricted to older individuals (ie, those ≥70 years) (Figure). In sensitivity analyses, our results were robust to changes in RRR, with all scenarios remaining cost-effective.

**Table.** Net Cost per 10 000 Infections and Cost-effectiveness Ratio^a^

Scenario	Age, y				Net cost, million $^b^	Severe illnesses averted, No.	DALYs averted, No.	Incremental cost, $	
	50-59	60-69	70-79	≥80				Per severe illness averted^c^	Per DALY averted^c^
0	No REGEN-COV	No REGEN-COV	No REGEN-COV	No REGEN-COV	[Reference]	[Reference]	[Reference]	[Reference]	[Reference]
1	None	None	None	T	–0.08	20	38	−4100^d^	–2100^d^
2	None	None	T	T	–0.10	46	66	–2200^d^	–1600^d^
3	None	T	T	T	–0.34	99	161	–3400^d^	–2100^d^
4	T	T	T	T	0.17	135	198	1200	800
5	None	None	None	P/T	0.11	23	45	4500	2300
6	None	None	T	P/T	0.08	50	73	1700	1100
7	None	T	T	P/T	–0.15	103	168	–1500^d^	–900^d^
8	T	T	T	P/T	0.35	139	205	2500	1700
9	None	None	P/T	P/T	0.50	54	78	9200	6400
10	None	T	P/T	P/T	0.26	107	173	2400	1500
11	T	T	P/T	P/T	0.77	143	209	5400	3700
12	None	P/T	P/T	P/T	1.11	117	190	9700	6000
13	T	P/T	P/T	P/T	1.64	153	226	10 700	7200
14	P/T	P/T	P/T	P/T	3.10	159	233	19 500	13 300

Abbreviations: DALY, disability-adjusted life-year; P/T, REGEN-COV is given as postexposure prophylaxis if exposed to an infected household member and for treatment if infected through non-household transmission; T, REGEN-COV is given to treat patients who are infected.

^a^ In US dollars (converted from Singapore dollars).

^b^ Numbers are rounded to the nearest $10 000.

^c^ Numbers are rounded to the nearest $100.

^d^ Cost-saving outcome (ie, better outcomes at lower cost).

**Figure.**
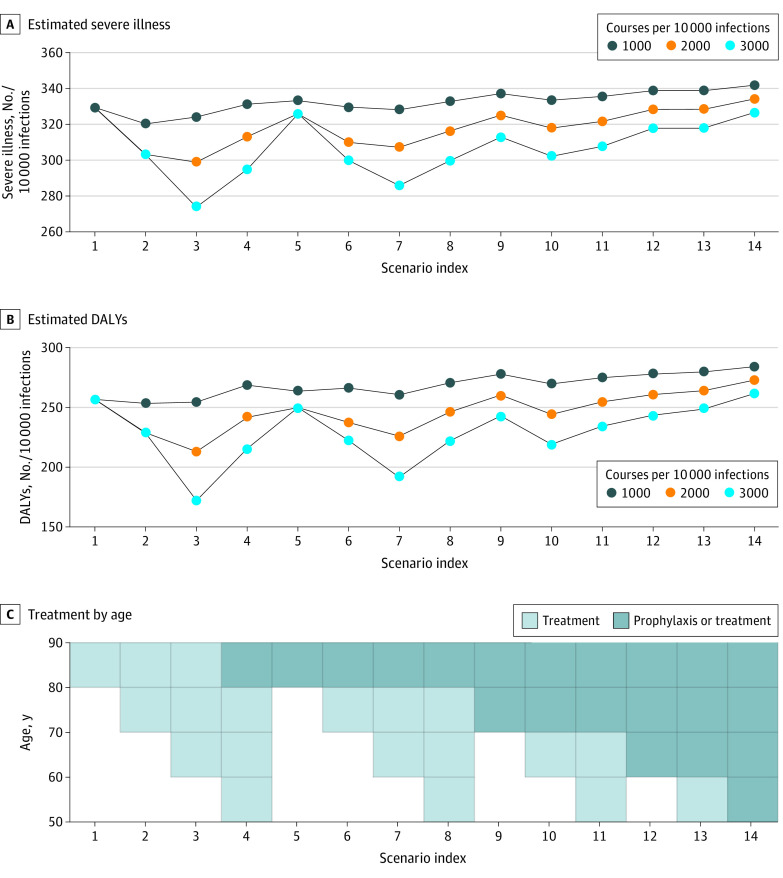
Estimated Number of Disability-Adjusted Life-Years (DALYs) and Severe Illnesses per 10 000 Infections Individuals of a given age group were assumed to receive REGEN-COV for treatment only (light gray) if infected or as postexposure prophylaxis if exposed to an infected household member or for treatment if infected through nonhousehold transmission (dark gray), assuming 1000, 2000, and 3000 courses of REGEN-COV were available for use in 14 scenarios. For each scenario, individuals were assumed to get REGEN-COV once, for prevention or treatment, given that those who had received postexposure prophylaxis owing to household contacts would not receive REGEN-COV again for treatment. In scenario 6, for example, indivduals aged 70 to 79 years would get REGEN-COV for treatment only, while those aged 80 to 89 years would get prophylaxis if exposed to household contacts or treatment if infected through nonhousehold transmission.

## Discussion

These findings suggest that in high-income settings, adults aged 60 years and older who are not fully vaccinated should be given priority to receive REGEN-COV for treating recently diagnosed COVID-19, particularly when supply is limited. It should be noted, however, that clinical trials on the use of REGEN-COV as PEP^2^ or for treatment^3^ were conducted before widespread circulation of the SARS-CoV-2 Delta and Omicron variants; therefore, a limitation of this analysis is that the estimated cost-effectiveness may be influenced if the efficacy of REGEN-COV differs by variant. Care must be taken to prevent the availability of monoclonal antibodies from deterring vaccination, which should remain the preferred means of preventing severe COVID-19.

## Notes

Supplement.
eAppendix.
**eFigure.** Strategies for Using REGEN-COV for Postexposure Prophylaxis (P) or Treatment (T) of COVID-19 **eTable 1.** Probabilities of COVID-19 Infection, Hospitalization, and Death by Vaccination Status and Age Group **eTable 2.** Cost and Length of Hospital Stay by Vaccination Status and Age Group **eTable 3.** Costs and Length of Hospital Stay by Vaccination Status and Age Group in Singapore Dollars
eReferences.
