# Supplementary material for: Estimated Health Outcomes and Costs Associated With Use of Monoclonal Antibodies for Prevention or Mitigation of SARS-CoV-2 Infections
Source: JAMA Netw Open. 2022 Apr 5;5(4):e225750. doi: 10.1001/jamanetworkopen.2022.5750 (PMC8984772; doi:10.1001/jamanetworkopen.2022.5750)
Supplement: Supplement. — eAppendix. eFigure. Strategies for Using REGEN-COV for Postexposure Prophylaxis (P) or Treatment (T) of COVID-19 eTable 1. Probabilities of COVID-19 Infection, Hospitalization, and Death by Vaccination Status and Age Group eTable 2. Cost and Length of Hospital Stay by Vaccination Status and Age Group eTable 3. Costs and Length of Hospital Stay by Vaccination Status and Age Group in Singapore Dollars eReferences. [file jamanetwopen-e225750-s001.pdf]

Supplemental Online Content

Park M, Tan KB, Vasoo S, Dickens BL, Lye D, Cook AR. Estimated health outcomes and costs associated with use of monoclonal antibodies for prevention or mitigation of SARS-CoV-2 infections. *JAMA Netw Open*. 2022;5(4):e225750. doi:10.1001/jamanetworkopen.2022.5750

eAppendix.
eFigure. Strategies for Using REGEN-COV for Postexposure Prophylaxis (P) or Treatment (T) of COVID-19
eTable 1. Probabilities of COVID-19 Infection, Hospitalization, and Death by Vaccination Status and Age Group
eTable 2. Cost and Length of Hospital Stay by Vaccination Status and Age Group
eTable 3. Costs and Length of Hospital Stay by Vaccination Status and Age Group in Singapore Dollars
eReferences.

This supplemental material has been provided by the authors to give readers additional information about their work.

© 2022 Park M et al. *JAMA Network Open.*

eAppendix.

Assumptions

Based on the findings from clinical trials, we assumed that REGEN-COV (casirivimab and imdevimab) reduces the risk of infection in household contacts (relative risk reduction, 66%)[1] and the risk of hospitalization or death related to COVID-19 (relative risk reduction, 70%)[2], while it shortens the duration of hospital stays by 2 days[2]. We presumed that if individuals who have received REGEN-COV for post-exposure prophylaxis become infected, their clinical outcomes are as if they were given REGEN-COV as treatment.

We assumed that REGEN-COV can be used for **post-exposure prophylaxis** in individuals who are at high risk of developing severe COVID-19, including those:

- who are not fully vaccinated,
- who have been exposed to SARS-COV-2 through a recently diagnosed household member, and
- who are at least 50 years of age.

Also, we assumed that REGEN-COV can be used for the **treatment** of COVID-19 in recently diagnosed individuals who are at high risk of developing severe COVID-19, including those:

- who are not fully vaccinated,
- who have been infected through non-household transmission (therefore had not been given REGEN-COV as post-exposure prophylaxis), and
- who are at least 50 years of age.

Estimating health impact of REGEN-COV

The total number of infections at baseline was estimated using the daily average number of community cases in Singapore from July 19 to August 18, 2021. We used daily reports available from the MOH website (COVID-19 Situation Report)[3] to extract the average proportion of household transmission as well as the proportion of patients by source of infection (household vs non-household), vaccination status (not fully vaccinated vs fully vaccinated), and age (50–59, 60–69, 70–79, 80+), making up 16 subgroups for infected individuals (2 infection sources × 2 vaccination status × 4 age groups) (**eFigure 1**). We then used the average proportion of patients in severe and critical condition, and those who died due to COVID-19 between May and August 2021 (**eTable 1**) to estimate the number of individuals in each of the subgroups without the use of REGEN-COV (status quo).

As post-exposure prophylaxis is being given to high-risk individuals who have been exposed to infected household members, we considered 8 more subgroups (4 age groups × 2 vaccination status) under household transmission to examine the effects of post-exposure prophylaxis in preventing the overall infection. To calculate the number of household contacts eligible for post-exposure prophylaxis, we used secondary attack rates for unvaccinated and vaccinated household contacts reported from a local study.[4]

Under 14 scenarios in which a fixed number of REGEN-COV is to be allocated and distributed to different groups of high-risk individuals, either for post-exposure prophylaxis or/and treatment (**eFigure 1**), we calculated changes in health outcomes, primarily the number of (i) severe cases requiring oxygen supplementation, (ii) critical cases admitted to ICU, and (iii) deaths due to COVID-19 averted as well as (iv) daily-adjusted life year (DALY), all per 10,000 cases.

The DALYs were calculated as the sum of the years of life lost due to premature mortality (YLLs) from COVID-19 and the years of healthy life lost due to disability (YLDs). YLLs were calculated as the estimated number of deaths from COVID-19 multiplied by the expected number of additional life-years for each age group. The expected number of additional life-years for each premature death was estimated based on the life expectancy table, which is publicly available from Statistics Singapore.[4] YLDs were calculated using disability weights of 0.133 and 0.655 for severe and critical COVID-19, respectively.[5] The average length of hospital stays for COVID-19 patients in both wards (for severe cases) and ICU (for critical cases and deaths) by age and by vaccination status were obtained from the National Centre for Infectious Diseases (NCID) of Singapore[5] (**eTable 2**) and used to approximate the duration of illness for YLDs. YLDs were calculated as the sum of YLDs for 8 subgroups (4 age groups x 2 vaccination status) for each severe and critical condition.

Based on the estimated number of eligible exposed and infected individuals under each scenario, we also examined changes in health outcomes including DALYs and the number of severe cases assuming there is a limited supply of REGEN-COV for use either for post-exposure prophylaxis or treatment (i.e., 1,000 – 3,000

© 2022 Park M et al. *JAMA Network Open.*

courses of REGEN-COV per 10,000 infections). Under limited supply, we assumed that the policy is maintained until supplies run out and thereafter the status quo (i.e., no REGEN-COV) is applied.

Estimating cost impact of REGEN-COV

The projected cost of REGEN-COV (including administration fees) per dose and the average cost of hospital stays for wards and ICU for COVID-19 patients were collated by the NCID and Ministry of Health of Singapore (**eTable 2**).[6] We assumed that those who receive REGEN-COV spend two days less in wards than those who do not[2] whereas for those with critical conditions there will be no difference in terms of the number of days spent in ICU between the two groups.

We estimated the cost of administrating REGEN-COV and hospitalization based on the number of severe and critical cases (including deaths) expected from each scenario. We then calculated net costs (per 10,000 cases) by subtracting the baseline cost without REGEN-COV (status quo) from the total cost with REGEN-COV under each scenario.

To note, all cost analyses were conducted using Singapore currency (**eTable 3**). Costs were then converted to US dollars based on the exchange rate (1 SGD = 0.742 USD) on February 3, 2022.

© 2022 Park M et al. *JAMA Network Open.*

					Household Transmission																		Non-Household Transmission							
					Household contacts								Infected								Infected							
	P/T by Age				Unvaccinated*				Vaccinated				**Unvaccinated**				Vaccinated				**Unvaccinated**				Vaccinated			
No.	50s	60s	70s	80+	50s	60s	70s	80+	50s	60s	70s	80+	50s	60s	70s	80+	50s	60s	70s	80+	50s	60s	70s	80+	50s	60s	70s	80+
0	No REGEN-COV				--	--	--	--	--	--	--	--	--	--	--	--	--	--	--	--	--	--	--	--	--	--	--	--
1	--	--	--	T	--	--	--	--	--	--	--	--	--	--	--	T	--	--	--	--	--	--	--	T	--	--	--	--
2	--	--	T	T	--	--	--	--	--	--	--	--	--	--	T	T	--	--	--	--	--	--	T	T	--	--	--	--
3	--	T	T	T	--	--	--	--	--	--	--	--	--	T	T	T	--	--	--	--	--	T	T	T	--	--	--	--
4	T	T	T	T	--	--	--	--	--	--	--	--	T	T	T	T	--	--	--	--	T	T	T	T	--	--	--	--
5	--	--	--	P/T	--	--	--	P	--	--	--	--	--	--	--	--	--	--	--	--	--	--	--	T	--	--	--	--
6	--	--	T	P/T	--	--	--	P	--	--	--	--	--	--	T	--	--	--	--	--	--	--	T	T	--	--	--	--
7	--	T	T	P/T	--	--	--	P	--	--	--	--	--	T	T	--	--	--	--	--	--	T	T	T	--	--	--	--
8	T	T	T	P/T	--	--	--	P	--	--	--	--	T	T	T	--	--	--	--	--	T	T	T	T	--	--	--	--
9	--	--	P/T	P/T	--	--	P	P	--	--	--	--	--	--	--	--	--	--	--	--	--	--	T	T	--	--	--	--
10	--	T	P/T	P/T	--	--	P	P	--	--	--	--	--	T	--	--	--	--	--	--	--	T	T	T	--	--	--	--
11	T	T	P/T	P/T	--	--	P	P	--	--	--	--	T	T	--	--	--	--	--	--	T	T	T	T	--	--	--	--
12	--	P/T	P/T	P/T	--	P	P	P	--	--	--	--	--	--	--	--	--	--	--	--	--	T	T	T	--	--	--	--
13	T	P/T	P/T	P/T	--	P	P	P	--	--	--	--	T	--	--	--	--	--	--	--	T	T	T	T	--	--	--	--
14	P/T	P/T	P/T	P/T	P	P	P	P	--	--	--	--	--	--	--	--	--	--	--	--	T	T	T	T	--	--	--	--

Prophylaxis (Household contacts columns); Treatment (Infected columns)

eFigure. Strategies for Using REGEN-COV for Postexposure Prophylaxis (P) or Treatment (T) of COVID-19

© 2022 Park M et al. *JAMA Network Open.*

eTable 1. Probabilities of COVID-19 Infection, Hospitalization, and Death by Vaccination Status and Age Group

	Unvaccinated				Vaccinated			
	50–59	60–69	70–79	80+	50–59	60–69	70–79	80+
**Proportions:**								
…household transmission[3]	63%	63%	63%	63%	63%	63%	63%	63%
…age and vaccination[3]	4%	2%	1%	1%	15%	12%	3%	1%
…infected by HH index[4]	26%	26%	26%	26%	11%	11%	11%	11%
…mild[a]	86%	68%	65%	45%	92%	99%	98%	92%
…severe[a]	12%	23%	25%	35%	9%	1%	1%	5%
…critical[a]	2%	6%	7%	5%	0%	0%	1%	1%
…severe, critical or dying[7]	14%	32%	35%	55%	9%	1%	2%	8%
…critical or dying[7]	3%	9%	9%	19%	0%	0%	1%	3%
…dying[7]	0%	3%	3%	15%	0%	0%	0%	1%

[a] Derived from other parameters

eTable 2. Cost and Length of Hospital Stay by Vaccination Status and Age Group

	Unvaccinated				Vaccinated			
	50–59	60–69	70–79	80+	50–59	60–69	70–79	80+
**Cost per person (US$):**								
Intervention:								
…REGEN-COV	2,326	2,326	2,326	2,326	NA	NA	NA	NA
…Administration (subcutaneous injection)	35	35	35	35	NA	NA	NA	NA
Hospitalization:								
…Wards (per day)[6]	329	329	329	329	329	329	329	329
…ICU (per day)[6]	2,099	2,099	2,099	2,099	2,099	2,099	2,099	2,099
**Average length of stays (ALOS):**								
…Wards (per day)[5]	19	15	25	25	13	13	15	16
…ICU (per day)[5]	10	19	7	5	5	3	6	13
…Reduction (days)[2]	2	2	2	2	2	2	2	2

© 2022 Park M et al. *JAMA Network Open.*

eTable 3. Costs and Length of Hospital Stay by Vaccination Status and Age Group in Singapore Dollars

	**Unvaccinated**				**Vaccinated**			
	50–59	60–69	70–79	80+	50–59	60–69	70–79	80+
**Cost per person (S$):**								
Intervention:								
…REGEN-COV	3,156	3,156	3,156	3,156	NA	NA	NA	NA
…Administration (subcutaneous injection)	48	48	48	48	NA	NA	NA	NA
Hospitalization:								
…Wards (per day)[6]	447	447	447	447	447	447	447	447
…ICU (per day)[6]	2,848	2,848	2,848	2,848	2,848	2,848	2,848	2,848
**Average length of stays (ALOS):**								
…Wards (per day)[5]	19	15	25	25	13	13	15	16
…ICU (per day)[5]	10	19	7	5	5	3	6	13
…Reduction (days)[2]	2	2	2	2	2	2	2	2

© 2022 Park M et al. *JAMA Network Open.*

eReferences.

1. O'Brien MP, Forleo-Neto E, Musser BJ, et al. Subcutaneous REGEN-COV Antibody Combination to Prevent Covid-19. *N Engl J Med*. Sep 23 2021;385(13):1184-1195. doi:10.1056/NEJMoa2109682
2. Weinreich DM, Sivapalasingam S, Norton T, et al. REGEN-COV Antibody Combination and Outcomes in Outpatients with Covid-19. *N Engl J Med*. Dec 2 2021;385(23):e81. doi:10.1056/NEJMoa2108163doi:10.1101/2021.05.19.21257469
3. Ministry of Health of Singapore. COVID-19 Situation Report. https://www.moh.gov.sg/covid-19/testing/situation-report-pdf
4. Ng OT, Koh V, Chiew CJ, et al. Impact of Delta Variant and Vaccination on SARS-CoV-2 Secondary Attack Rate Among Household Close Contacts. *Lancet Regional Health Western Pacific*. 20213(in print)
5. Cuschieri S, Calleja N, Devleesschauwer B, Wyper GMA. Estimating the direct Covid-19 disability-adjusted life years impact on the Malta population for the first full year. BMC Public Health. 2021 Oct 9;21(1):1827. doi: 10.1186/s12889-021-11893-4.
6. National Centre for Infectious Diseases. LOS of COVID-19 patients admitted to NCID in 2021 by age group and vaccination status (as of 29 Aug 2021) (Unpublished). 2021.
7. Ministry of Health of Singapore. Cost of hospital (GW/ ICU) stay per COVID-19 patient per day (Unpublished). 2021.
8. Ministry of Health of Singapore. Analysis of severity amongst vaccinated and unvaccinated COVID-19 patient (Unpublished). 2021.

© 2022 Park M et al. *JAMA Network Open.*
